# Endolysosomal Ca^2+^ Signaling in Cancer: The Role of TPC2, From Tumorigenesis to Metastasis

**DOI:** 10.3389/fcell.2019.00302

**Published:** 2019-12-04

**Authors:** Abeer F. Alharbi, John Parrington

**Affiliations:** ^1^Department of Pharmacology, University of Oxford, Oxford, United Kingdom; ^2^Department of Pharmaceutical Sciences, College of Pharmacy, King Saud Bin Abdulaziz University for Health Sciences, Riyadh, Saudi Arabia

**Keywords:** endolysosomal, calcium, Ca^2+^ signals, TPC2, cancer

## Abstract

Ca^2+^ homeostasis is dysregulated in cancer cells and affects processes such as tumorigenesis, angiogenesis, autophagy, progression, and metastasis. Emerging evidence has suggested that endolysosomal cation channels sustain several cancer hallmarks involving proliferation, metastasis, and angiogenesis. Here, we investigate the role of TPC1-2, TRPML1-3, and P2×4 in cancer, with a particular focus on the role of TPC2 in cancer development, melanoma, and other cancer types as well as its endogenous and exogenous modulators. It has become evident that TPC2 plays a role in cancer; however, the precise mechanisms underlying its exact role remain elusive. TPC2 is a potential candidate for cancer biomarkers and a druggable target for future cancer therapy.

## Introduction

Cancer is a major public health problem and is one of the leading causes of death worldwide. It is a disease associated with abnormal tissue growth that involves dysregulation of fundamental cellular processes such as apoptosis, proliferation, differentiation, autophagy, and migration. According to Cancer Research United Kingdom, someone in the United Kingdom dies from cancer every four minutes (Cancer Statistics for the UK, 2018). The treatment options for cancer are surgery, radiotherapy, chemotherapy, immunotherapy, targeted therapy, hormone therapy, and stem cell transplants. Significant advances have been made in the treatment of both solid and hematologic malignancies, thus giving many patients the hope of a cure. However, current therapies are associated with severe side effects and a low response rate. Therefore, new tumor hallmarks and mechanisms of tumorigenesis must be identified to develop new treatment strategies.

Calcium is a ubiquitous secondary messenger, and intracellular calcium ions (Ca^2+^) play a significant role in various physiological processes. They act as regulators of gene transcription and cell proliferation, differentiation, migration, and death (Cui et al., 2017). Alterations of Ca^2+^ signaling via changes in the expression or post-translational modification of Ca^2+^ transporters and Ca^2+^ binding proteins play a role in cancer development from tumor initiation to migration as well as in chemotherapy resistance (Xu et al., 2018). Accumulated data have shown that Ca^2+^ homeostasis is dysregulated in cancer cells and has an impact on processes such as tumorigenesis, angiogenesis, autophagy, progression, and metastasis (Cui et al., 2017). Therefore, the regulation of spatiotemporal Ca^2+^ signals in the form of waves or oscillations has emerged as a topic of research relevant to the diagnosis and treatment of cancer. Ironically, the ubiquity of Ca^2+^ signaling pathways in normal cellular functions makes it challenging to specifically target the molecular components of Ca^2+^ signaling pathways, as they are also fundamental for normal vital physiological processes. One relevant strategy in this respect would be to target a specific intracellular Ca^2+^ store. The endolysosomal system has recently become recognized as an important new type of intracellular Ca^2+^ store (Morgan et al., 2011). The role of lysosomal Ca^2+^ has been demonstrated in cell growth and cancer (Parrington et al., 2015). The major endolysosomal Ca^2+^ channels are two-pore channels (TPCs), transient receptor potential cation channels, mucolipin subfamily (TRPML), and P2×4 adenosine triphosphate (ATP)-activated cation channels.

Two-pore channel 2 (TPC2 or TPCN2) is a eukaryotic ion channel located in the lysosome. The release of Ca^2+^ from this channel is regulated by the second messengers nicotinic acid adenine dinucleotide phosphate (NAADP) and phosphatidylinositol 3,5-bisphosphate (PI(3,5)P2). However, much remains to be established about how TPC2 interacts with NAADP. Additionally, Jha et al. (2014) have discovered novel regulators of TPC2: Mg^2+^ and the mitogen activated protein kinases (MAPKs) c-jun N-terminal kinase (JNK) and P38. Importantly, it has been found that the mechanistic target of rapamycin complex 1 (m-TORC1) controls lysosomal Ca^2+^ release via TPC2 (Ogunbayo et al., 2018). Recently, a growing body of literature has identified potential links between TPC2 and cancer. The cellular mechanisms underlying the regulation of Ca^2+^ signaling via TPC2 in different cancer cells and pathophysiological processes have not yet been deeply investigated, and research in this area is still in its infancy. In this article, we review the role of endolysosomal cation channels in cancer, particularly the association between TPC2 and key pathophysiological processes involved in cancer development, to uncover the roles of TPC2 in different stages of cancer from tumor initiation to tumor cell migration as well as its potential as a novel hallmark of tumors and a druggable target for future cancer therapy.

## Endolysosomal Ca^2+^ Channels and Cancer

The endolysosomal system involves lysosomes; early, late, and recycling endosomes; and autophagosomes. Cation channels located in the endolysosomal system consist of TPCs, TRPML, and ATP-regulated P2×4 channels. A high Ca^2+^ concentration of ∼600 μM is found in the lysosomal lumen (Lloyd-Evans et al., 2008). Several studies have highlighted the difference between the functions of lysosomes in cancer cells compared to those in normal cells (Hämälistö and Jäättelä, 2016). The lysosome-cancer link has been established in different fundamental processes involving tumor progression and metastasis of cancer and chemotherapy resistance (Fehrenbacher and Jäättelä, 2005; Dozynkiewicz et al., 2012; Zhitomirsky and Assaraf, 2016).

Transient receptor potential cation channels include TRPML1, TRPML2, and TRPML3, all of which are located in the endolysosomal system. TRPML-mediated Ca^2+^ signaling plays a role in vesicular trafficking events involved in autophagy and lysosomal exocytosis (Li et al., 2018). Phosphatidylinositol 3,5-bisphosphate (PI(3,5)P2) activated lysosomal Ca^2+^ releases from TRPML in a pH-dependent manner for TRPML1 and TRPML3 (Xu et al., 2007; Li et al., 2017; Zhou et al., 2017). Besides, (PI(3,5)P2) reactive oxygen species (ROS) triggers Ca^2+^ signals via TRPML1; both are known as endogenous agonists (Li et al., 2018). The importance of targeting TRPML channels has led to the development of several synthetic modulators such as mucolipin synthetic agonist 1 (ML-SA1), MK6-83, TRPML synthetic inhibitor (ML-SI), and ML-SI3 which pharmacologically modulates TRPML channels, whereas ML2-SA1, SN-2, SF-21, and SF-22 activate TRPML2, TRPML3, TRPML2 and TRPML3, and TRPML1 and TRPML3, respectively (Grimm et al., 2010, 2014a; Chen et al., 2014; Li et al., 2018; Plesch et al., 2018; Zhang et al., 2019).

TRPML1 acts as a regulator of mTOR and TEFB signaling (Li et al., 2016). Ca^2+^ signaling through TRPML1 modulates mTORC1 activity, TFEB localization, and autophagosome-lysosome fusion to control autophagy (Di Paola et al., 2018). The dysregulation of autophagy is associated with carcinogenesis and chemoresistance (Owusu-Brackett et al., 2019). mTOR is involved in PI3K/AKT/mTOR signaling, which plays a crucial role in the pathogenesis of cancer (Li et al., 2016). A recent study illustrated that the dysregulation of TEFB expression or activity is correlated with pancreatic cell proliferation (Marchand et al., 2015). TFEB acts as a positive regulator of post-ischemic angiogenesis via AMPKα signaling and autophagy in endothelial cells. Furthermore, TFEB has been found to upregulate TRPML1 expression at the mRNA level and to bind to its promoter (Fan et al., 2018). According to these data, we can infer that mucolipin-1 (*MCOLN1*) might act as a mediator of tumor angiogenesis. *MCOLN1* is upregulated in triple-negative breast cancer cell lines, and it mediates tumor development via the mTORC1 pathway and lysosomal ATP release (Xu et al., 2019). TRPML-1 modulation either by genetic downregulation or by pharmacological inhibition using ML-S11 was shown to hamper tumor growth and impair cell migration and invasion (Xu et al., 2019). Similarly, *MCOLN1* is upregulated in HRAS-expressing cancer cells and plays a role in *HRAS*-positive-tumor proliferation. Low expression of TRPML-1 was correlated with significantly enhanced survival in HRAS mutant patients with bladder urothelial carcinoma or head and neck squamous cell carcinoma (Jung et al., 2019). A strong association has been shown between loss of/reduced TRPML-1 mRNA-expression and poor survival, revealing the potential application of *MCOLN1* as a prognostic biomarker in glioblastoma patients (Morelli et al., 2019). TRPML2 overexpression promotes glioma cell proliferation (Morelli et al., 2016). TRPML3 is involved in biological processes including trafficking, autophagy, and endocytosis (Grimm et al., 2017). TRPML-mediated lysosomal Ca^2+^ release sustains several cancer hallmarks, including proliferation and autophagy. The protumorigenic function of TRPML warrants further investigation and is a candidate for a novel therapeutic approach to intercepting the pathological process related to cancer development.

Adenosine triphosphate-gated P2×4 cation channels are located in the late endosome, lysosome, and plasma membranes (Li et al., 2018). Luminal ATP and lysosomal pH modulate Ca^2+^ release from P2×4 (Li et al., 2018). P2×4-mediated lysosomal Ca^2+^ release leads to lysosomal membrane fusion in a calmodulin-dependent manner (Yang et al., 2019). Accumulated data show that P2×4 plays a principal role in cancer-associated pain, suggesting P2×4 modulation as a potential strategy for developing new treatments to alleviate cancer-related pain (Franceschini and Adinolfi, 2014; Yan et al., 2019).

**FIGURE 1:**
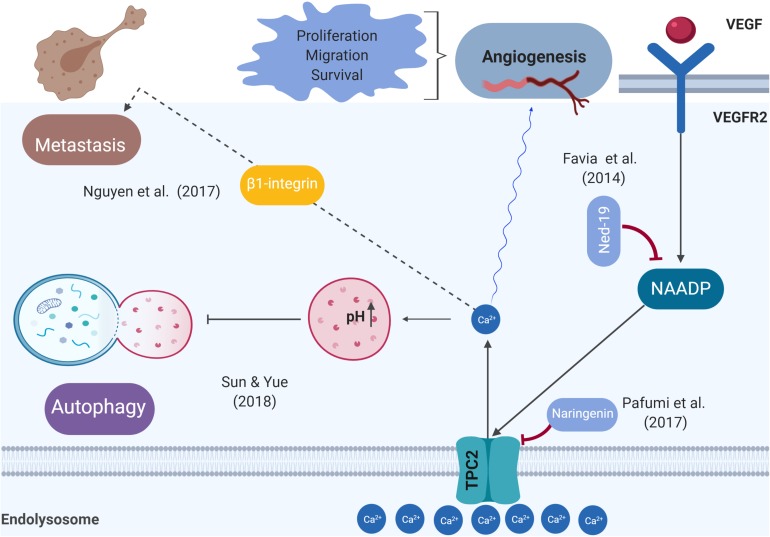
Schematic representation of the role of TPC2 in the pathophysiological processes related to cancer. Previous studies demonstrated that the VEGFR2/NAADP/TPC2/Ca^2+^ signaling pathway is critical for VEGF-induced angiogenesis *in vitro* and *in vivo*.

Two-pore channels, including TPC1 and TPC2, are ubiquitously expressed in the endolysosomal system of mammalian cells. NAADP and phosphatidylinositol 3,5-bisphosphate (PI(3,5)P2) mediate the release of lysosomal Ca^2+^ (Li et al., 2018). TPCs modulate membrane trafficking, which plays a role in several physiological processes involving endocytosis, exocytosis, autophagy, and viral infection (Li et al., 2018). Recent evidence has linked TPCs to cancer development and progression, suggesting that TPCs are druggable targets that can interfere with tumorigenesis, angiogenesis, and metastasis (Brailoiu et al., 2009; Favia et al., 2014; Jha et al., 2014; Pafumi et al., 2017; Sterea et al., 2018). Other findings have highlighted the link between TPC1 expression dysregulation and tumorigenicity, demonstrating that TPC1 transcripts were approximately three to eightfold higher than TPC2 ones in the SKBR3 human breast cancer cell line (Brailoiu et al., 2009). Another recent study showed that NAADP-mediated TPC1 lysosomal calcium release triggers ERK and the PI3K/AKT signaling pathways, thereby promoting the proliferation of metastatic colorectal cancer (mCRC) cells. In this regard, TPC1 might be a suitable biological target that may reduce cancer growth in mCRC patients, warranting further investigation *in vivo* (Faris et al., 2019). Figure 1 summarizes the likely roles of TPC2, from tumor initiation to tumor cell migration.

## TPC2, Tumorigenesis, and Tumor Progression Involving Angiogenesis

Ca^2+^ signaling pathways are emerging as playing a key role in tumor initiation, involving increased proliferation and apoptosis resistance, two of cancer’s hallmarks (Xu et al., 2018). Alterations of Ca^2+^ homeostasis may behave more as a driver than as a passenger in tumorigenesis (Cui et al., 2017). TPC2 is found on chromosome 11, region 13.2 (*TPCN2*, NCBI Gene ID: 219931) (TPCN2 two pore segment channel 2 [Homo sapiens (human)], 2018). Data from several sources have shown that 11q13-q14 amplifications are associated with cancer (Wilkerson and Reis-Filho, 2012). This finding is consistent with that of Huang et al. (2018), who observed that TPC2 is overexpressed in oral squamous cell carcinoma cell lines (Huang et al., 2018). These findings raise intriguing questions regarding the roles of TPC2 and a number of other nearby genes as drivers of oncogenesis. Recently, Wei Sun et al. drew attention to the role of TPC2 in cancer cell proliferation. They showed that TPC2 knockdown cells proliferated more slowly than the control or TPC2 overexpressing cells (Sun and Yue, 2018). It is therefore likely that such connections exist between TPC2 and tumor growth. Further research should be undertaken to investigate the possibility that TPC2 acts as a driver to enable a cell to reach the tumorigenic state and as a promoter of tumor growth. Many unanswered questions remain about how precisely TPC2 regulates tumorigenesis and tumor progression at the molecular level. Angiogenesis is a process crucial for cancer progression and a key step in the transition of a tumor’s state from benign to malignant, whereby endothelial cells give rise to new blood vessels. Tumor cells release vascular endothelial growth factor (VEGF) that triggers signal transduction to facilitate Ca^2+^ signaling, leading to endothelial cell proliferation (Fehrenbacher and Jäättelä, 2005). A previous study demonstrated that the VEGFR2/NAADP/TPC2/Ca^2+^ signaling pathway is critical for VEGF-induced angiogenesis *in vitro* and *in vivo* (Favia et al., 2014). *In vivo* vascularization meditated by VEGF was abolished by Ned-19 (a TPC2 antagonist) and also in TPC2 knockout mice (Favia et al., 2014). A recent study revealed that naringenin inhibits VEGF-induced angiogenesis via TPC2 (Pafumi et al., 2017). An implication of this is the possibility that targeting TPC2 to develop anti-angiogenics is a strategy for cancer treatment.

## TPC2 and Autophagy in Cancer

Autophagy captures, degrades, and recycles proteins and organelles in lysosomes to maintain metabolism and cellular homeostasis (Santana-Codina et al., 2017). Autophagy plays a dual role in cancer: it constrains tumorigenesis in normal tissue and promotes tumorigenesis in cancer tissue by overcoming microenvironmental stresses and conferring resistance to chemotherapy (Mathew et al., 2007). Previous studies have shown that intracellular Ca^2+^ signaling regulates both basal and induced autophagy (Kondratskyi, 2013). A much-debated question is whether TPC2 promotes or hampers autophagy at an early or late stage of the autophagic process (Pereira et al., 2011; Kayala et al., 2012; García-Rúa et al., 2016; Bootman et al., 2018). Starvation was shown to increase autophagy flux and to exacerbate autophagosome accumulation in response to colchicine (a microtubule inhibitor) in the skeletal muscle of TPC2 knockout mice (Lin et al., 2015). In contrast, a recent study investigating the role of TPC2 in the autophagy of cancer cells reported that overexpression of TPC2 in the Hela human cervical cancer cell line and in a 4T1 mouse breast cancer cell line diminished autophagosomal–lysosomal fusion, resulting in the accumulation of microtubule-associated protein light chain 3 (LC3)-II and syntaxin 17 (STX17)-positive autophagosomes (Sun and Yue, 2018). Overall, these findings indicate that TPC2 mediates autophagy. Therefore, the pharmacological modulation of TPC2 to inhibit autophagy is an attractive therapeutic strategy for enhancing cancer therapy, particularly in advanced stages, because there is a lack of drugs that diminish autophagy and exhibit selective antitumor effects. However, a better understanding of the molecular mechanisms underlying the TPC2 modulation of autophagy and the identification of related, novel, and specific downstream targets for drugs require further investigation.

## TPC2, Metastasis, and Cancer Invasion

Metastatic cancer causes the majority of cancer-related mortalities, accounting for ∼90% of such deaths (Chaffer and Weinberg, 2011). Tumor metastases occur only after several stages: the key steps are the loss of cell-cell connections, transformation of primary cancer cells into migratory mesenchymal cells, and invasion of tumor cells (Xu et al., 2018). Chaffer and Weinberg (2011) discussed metastasis and summarized it as a two-phase process. The first phase involves the ability of cancer cells to physically translocate to the “site of dissemination,” and the second phase is represented by the primary tumor cells gaining the ability to colonize secondary sites and develop metastatic lesions (Chaffer and Weinberg, 2011). An *in vitro* model using siRNA to silence TPC1 or TPC2 and pharmacologic inhibition of TPC activity with Ned-19 or tetrandrine to investigate the role of TPCs in cancer migration indicated that the adhesion and formation of leading edges as well as migration of cancer cells were reduced (Nguyen et al., 2017). When TPC2 function was impaired by siRNA, Ned-19, or tetrandrine, this minimized the formation of lung metastases in an *in vivo* mouse model of mammary cancer cells (Nguyen et al., 2017). These results highlight the requirement for TPC1 and TPC2 in two critical steps of metastasis formation: adhesion and migration. A possible explanation for these results may be that the disruption of TPC function is hindered by the trafficking of β1-integrin, which is a protein that is prominently involved in tumor migration. Its promigratory trafficking occurs via the endolysosomal system, leading to its accumulation in endocytic vesicles (Nguyen et al., 2017). Consistent with the literature, disturbances in Ca^2+^ homeostasis alter trafficking and lead to the fusion of endocytic vesicles (Ruas et al., 2010; Nguyen et al., 2017). Similarly, endolysosomal Ca^2+^ signaling plays a role in the migration of neural crest cells (Patel, 2018). To develop a full picture of the role of TPC2 in regulating metastasis, additional studies of the *in vivo* TPC2 knockout mouse model are needed. Targeting integrins or the downstream protein(s) that interacts with TPC2 may be a promising strategy for treating metastatic carcinomas.

## TPC2 in Melanoma

Melanoma is a type of skin cancer that emerges from pigmented melanocytes (Regad, 2013). Melanosomes are lysosomal organelles that synthesize melanin. Alterations in melanin synthesis increase a person’s risk of developing skin cancer (Bellono et al., 2016). Many studies have begun to provide links between TPC2, cellular pigmentation, and melanoma. Lipid phosphatidylinositol bisphosphate (PI(3,5)P2) mediates TPC2 activity, which acts as a regulator of pigmentation, by controlling melanosomal pH and membrane potential (Bellono et al., 2016). In Xenopus oocytes, TPC2 overexpression evoked pigmentation defects, and its interactivity with Rab GTPases underpinned NAADP-evoked Ca^2+^ signals (Lin-Moshier et al., 2014). Polymorphisms in the TPC2N gene were identified (rs35264875 and rs3829241) and were associated with a shift from brown to blond hair (Sulem et al., 2008). These two non-synonymous genetic variants were functionally characterized by endolysosomal patch-clamp techniques; this revealed that they were associated with a gain of TPC2 function by independent mechanisms (Chao et al., 2017). Therefore, it seems that TPC2 overexpression generally induced a decrease in melanin production and increased susceptibility to skin cancer.

Despite these promising results, further work is required to confirm the link between TPC2 and melanoma in mouse models and to reveal molecular mechanisms. However, the above findings suggest that TPC2 might represent a viable therapeutic target for the treatment of skin cancer.

## TPC2 in Other Cancer Types

In contrast to previous findings that had identified TPC2 overexpression as a potential risk factor for skin cancer, TPCN2 expression was found to be significantly associated with increased survival in bladder cancer (*P*-value = 3.56E^–02^). Further investigation is needed to investigate its clinical utility as a potential prognostic marker for bladder cancer (Shivakumar et al., 2017). Two TPCs (TPC1 and TPC2) were expressed in all malignant and benign breast cancer lines. Therefore, they are a feature of all breast cancer subtypes and not specific to one such subtype (Jahidin et al., 2016). The knockdown of TPC2 (although not of TPC1) significantly diminishes epidermal growth factor-induced vimentin expression in MDA-MB-468, a triple-negative breast cancer cell line. TPCN2 is reported to be one of the six gene signatures correlated with prostate cancer to predict postoperative biochemical recurrence (Li et al., 2019). The exploration of the links between TPC2 and different types of cancer requires further work to identify any significant genetic variants associated with such cancers. It would then be interesting to develop *in vitro* and *in vivo* models to examine the role of these TPCN2 mutations during tumor formation (involving neoangiogenesis) and cancer-cell migration as well as their clinical utility as diagnostic or prognostic biomarkers in different cancers.

## TPC2 Endogenous Agonists, Endogenous and Exogenous Inhibitors, and Interactomes

TPC2 endogenous agonists include (PI(3,5)P2) and NAADP, whereas torin-2 is an exogenous stimulator that mediates lysosomal Ca^2 +^ release via mTOR inhibition. The endogenous inhibitors are cytoplasmic ATP, mTOR, and Mg^2+^, whereas the synthetic inhibitors are Ned-19, naringenin, and tetrandrine (Pafumi et al., 2017; Li et al., 2018). A recent study was conducted for repurposing the screens of ∼1500 FDA-approved drugs, and it identified fluphenazine and raloxifene as TPC2 blockers (Penny et al., 2019). HCLS1-associated protein X-1 (Hax1), a negative regulator of autophagy and apoptosis, has been physically identified as a TPC2-interactor (Lam et al., 2013). Despite the importance of TPC2 interactomes, there remains a paucity of validated evidence about the proteins that interact with TPC2 to regulate its activity in different physiological processes. Annexin A (ANXA) 1-7, Ras-related protein Rab-7, and Ankyrin repeat domain-containing protein 27 (ANKRD27) are potential candidates for mediators of TPC2 function (Lin-Moshier et al., 2014; Krogsaeter et al., 2018). A recent review summarized how the identified TPC2 SNARE-interactomes mediate membrane fusion or vesical fusion (Krogsaeter et al., 2018). Soluble N-ethylmaleimide-sensitive factor attachment protein receptor (SNARE) proteins play a role in vesicle trafficking and membrane fusion (Han et al., 2017; Wang et al., 2017). TPC2 interacts with Q-SNARE proteins, involving syntaxin 6 (STX6), syntaxin 7 (STX7), syntaxin 12 (STX12), syntaxin 16 (STX16), syntaxin 18 (STX18), VTI1B, and SNAP23 (Grimm et al., 2014b; Lin-Moshier et al., 2014). VAMP2 and VAMP3 are identified as R-SNARE-TPC2 interactors (Huttlin et al., 2017; Krogsaeter et al., 2018). Further research to reveal the complex interplay of SNARE-TPC2 interactions is strongly recommended. Moreover, investigating the interaction between these candidates and TPC2 and then validating it by co-immunoprecipitation and other approaches would be a fruitful and novel area for further research.

## Closing Remarks

In summary, with growing evidence linking endolysosomal Ca^2+^ signaling pathways, particularly TPC2, to cancer, this review reveals the role of such pathways, especially TPC2, in cancer development from tumor initiation to metastasis. The mechanisms that underpin the role of TPC2 in different fundamental processes of cancer and in different types of tumors are not fully understood. TPC2 modulation either by genetic approaches such as siRNA or CRISPR/Cas9, or pharmacologically, is a promising therapeutic target to treat cancer. However, there is a potential for off-target effects arising from the ubiquity of TPC2. Therefore, discovering proteins that specifically interact with TPC2 in cancer tissue instead of targeting TPC2 itself is another approach for drug development that could overcome this effect. Although it has become evident that TPC2 plays a role in cancer, the precise mechanisms underlying the action of TPC2 and its involvement in cancer remain elusive and unclear. Further research in this field would be of great help in expanding our knowledge of TPC2’s action at the molecular level.

## Author Contributions

AA has contributed to the mini-review-research question and designing the study, data collection, and interpretation, writing manuscript, designing and creating the figure, and addressing the reviewers’ comments. JP has revised the manuscript.

## Conflict of Interest

The authors declare that the research was conducted in the absence of any commercial or financial relationships that could be construed as a potential conflict of interest.
